# Experimental study into the effects of pitch and coning angles on the flight performance of the natural samara

**DOI:** 10.1098/rsos.240758

**Published:** 2024-09-30

**Authors:** Byung Kwon Jung, Djamel Rezgui

**Affiliations:** ^1^ School of Civil, Aerospace and Design Engineering, University of Bristol, Bristol BS8 1TR, UK

**Keywords:** leading-edge vortex, rotary seed, maple seed, flight test, wind tunnel

## Abstract

Using their light wing and through the use of a leading-edge vortex (LEV), autorotating samaras can generate high lift while descending at extremely low speeds. But the flight performance of the samara, with respect to the wide design envelope, is still not well understood. Therefore, this paper aims to experimentally assess how the flight performance of three natural samara wings varies with differing wind speeds and flight conditions. The tests were conducted within a vertical wind tunnel and a novel rig was devised to effectively measure the vertical thrust and rotational rate of the autorotating samara at near frictionless conditions. Furthermore, a bespoke hub was implemented to control the coning and pitch angles of the samara wing. The tests generated a novel and comprehensive set of experimental data of autorotating samaras with changing wind speed, coning and pitch angles. The results also revealed that coning angles between 5 and 15 degrees can increase the vertical thrust produced by the samara by up to a maximum of 9.6% . Additionally, it was found that samaras operate at extremely low pitch angles between −0.7 and −2.6 degrees to maximise their thrust, even though the conditions are close to the autorotational stability boundary.

## 1. Introduction

**Figure 1.**
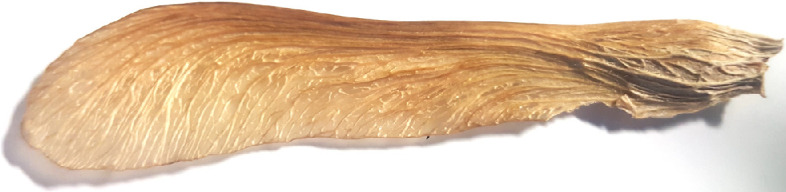
Photograph of a silver maple (*Acer saccharinum*).

Samara is a name given to a type of seed where the dry fruit is enveloped by a papery wing-like tissue [1], as shown in figure 1. Normally on a windy day, mature samara seeds will detach from the tree and, with their single or multiple wings, will achieve flight by rotating their wings about like a helicopter. Thus, samaras are often referred to as winged or rotary seeds [2]. The spinning wing, analogous to a helicopter rotor blade, produces a large aerodynamic thrust force that opposes the weight, and this helps the seed to descend slowly at a constant speed towards the ground [3,4]. The slow descent prolongs the flight time of the samara, thereby increasing the chance that the seed will be caught by an up-gust or side wind. Consequently, the likelihood for the seed ending up further away from the parent tree is increased, which is a crucial factor for the juvenile samara seeds in achieving successful germination [5,6].

All samara seeds are different, not only across family and genus but even within the same species [7]. Each seed matures to a specific size and mass, and develops a particular wing shape, surface roughness and inertial distribution, all of which provide distinct aerodynamic and flight characteristics of its own [8,9]. Therefore, the perpetual spinning flight, known as autorotation, is unique in performance for each and every samara.

Over the years, the aerodynamic performance of samaras has been most commonly assessed via the droptest method. In this method, a samara is released from a sufficient height above the ground within a calm (or stagnant) air setting, and the ensuing descent is recorded in real time via a single or multiple sets of high-speed cameras [3,10–12]. The samara, upon release, will first undergo rapid changes in the dynamics as the rotation initiates; this is known as the transition stage. However, after a few split seconds, it will enter steady autorotation as all the forces, moments and torque acting on and produced by the seed come into balance. This is known as the stable autorotation stage [13]. It is at this stable autorotational stage that the steady flight characteristics of the samara, collectively known as the flight parameters, are extracted.

**Figure 2.**
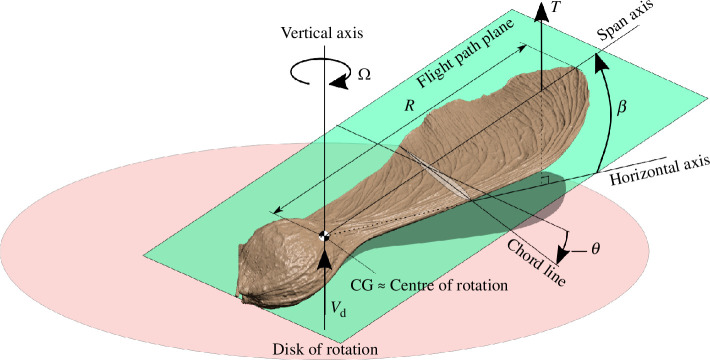
Flight parameters of the autorotating samara in vertical descent. $V_{\text{d}}$ is the descent speed, Ω is the rotational rate, β is the coning angle, θ is the pitch angle, and T is the vertical thrust produced by the samara.

The flight parameters of the autorotating samara are as follows: $V_{\text{d}}$ is the descent or the sinking speed of the seed, Ω is the rotational rate about the axis of rotation, β is the coning angle, i.e. the angle between the span and the horizontal axis and θ is the pitch or the feathering angle; the angle between the cross-sectional chordline of the samara and the chord axis within the flight path plane, as illustrated in figure 2. Interestingly, many droptest analyses have suggested that samaras belonging to the same genus operate with similar values in the flight parameters. For example, the most common singled-winged maple samaras (*Acer* species) vertically descend at $V_{\text{d}}=$ 0.75 ∼ 1.2 m s^−1^, autorotate at Ω of 1000 ∼ 1200 rpm, operate with a β of 10 °∼ 20 ° and descend with a θ that range from −1° to −4° within a calm air setting [8,14,15].

Such droptest flight data have been invaluable in understanding the flight mechanics of samaras, but they also lead to several important questions encompassing samara flight, one of which was ‘How are samaras able to descend at extremely slow speeds, given that they have small wings?’. This particular paradox only came to light with the advancement in the flow visualisation technique, whereby particle image velocimetry (PIV) has allowed the discovery of a special flow mechanism known as the leading-edge vortex (LEV) [16]. The vortex, which forms on the leading edge of the winged seed, remains both stable and attached to the revolving wing, thereby generating enhanced circulation and creating a region of low pressure above the wing, which some argue that it creates an ‘extra lift’ [17,18]. However, more importantly, the LEV helps keep the flow attached even at extreme angles of attack of up to 70 ° and above. Owing to this ‘absence of stall’, the lift coefficient and lift can continually rise with the angle of attack, leading to greater lifting capabilities [19,20]. Following the discovery, detailed studies on the samara LEV have prevailed and to a certain extent have explained the high lift and slow descent observed in samara flight [21–23].

The LEV, however, is far from being the full story, since the flight performance of the samara is strongly dictated by its autorotative ability and unfortunately, studies on these topics have been fairly limited. The most in-depth experimental research related to the flight characteristics of autorotative seeds dates back to the 1990s, when Yasuda & Azuma built a series of styrene-foam samara wing models and tested them within a wind tunnel setting [24]. From the experiment, the authors were able to identify features of the natural samara that contributed to a low descent rate and favourable autorotative ability; these being heavy nut, the thick leading edge, the surface roughness of the wing and the camber at the wing root. These features, commonly observed for all single-winged samara species, were suggested to improve the flight aerodynamics; but more importantly, they helped push the chordwise centre of gravity (CG) to be located near the leading edge and spanwise CG to near the wing root. This natural CG location, more or less common for all single-winged rotary seeds, was said to be the critical factor in achieving optimal autorotation.

Recently, Kwon & Sohn [25] investigated in greater detail, the role of CG position on the flight characteristics of maple seeds. The authors conducted an in-depth sensitivity study by executing a multitude of six d.f. numerical simulations of a descending autorotative maple seed that had various spanwise and chordwise CG locations. The results suggested that as the seed’s chordwise CG moved towards the leading edge, the seed autorotated with increased Ω and with a more negative θ setting. Furthermore, a small change in the chordwise CG location and θ angle, led to a drastic change in $V_{\text{d}}$ , Ω , and β . On the other hand, the effects of moving the spanwise CG location had much lesser implications. Overall, this study proved to be extremely useful in establishing the interdependence of the seed’s mass distribution and flight parameters. However, whether the suggested rationale is reflective of real maple seeds still needs to be verified.

Furthermore, there exists another flight parameter that has been treated with less importance, which is the vertical thrust ( T ) of the autorotating seed. This particular parameter has been underexplored due to its simplicity—in perfectly calm air conditions (i.e. in a droptest setting), the descending autorotating samara will produce a vertical thrust that is equal to its weight. In the real world, however, the samara’s flight envelope is far greater as the airflow is not stagnant. Hence, in most cases, the samara will produce a vertical thrust that is not equal to its weight and the seed will experience acceleration or deceleration in its descent speed. Therefore, it is important to measure the thrust produced by the autorotating samaras within a wider flight envelope. Recognizing this, the current authors have successfully measured the vertical thrust and rotational rate of the autorotating samara at a range of wind speeds within a wind tunnel setting. However, the focus of the previous research conducted was on creating a numerical model of the autorotating samara and defining the sectional lift and drag coefficients of the samara [20,26]. Hence, the wind tunnel test data generated were either confined to a single samara [26] or had tight restrictions in the coning and pitch angles [20].

Additionally, there is a great demand for a more comprehensive set of samara experimental test data. Over the recent years, numerous samara inspired micro air vehicles [27–30], wind turbines [31–33] and space capsules [34,35] have been introduced, all of which hope to make use of the high lifting capability that samara seeds provide. These systems, however, were designed without the full understanding of samara flight parameters. For instance, the reason ‘why samaras choose to operate with a specific range of coning and pitch angles?’ remains ambiguous. To solve this enigma, it is evident that more in-depth experimental tests of samara seeds are necessary, especially in exploring the influence of flight parameters on the flight performance of the seed.

On that account, the present study aims to generate a novel and complete set of samara experimental test data, with a focus on exploring the effects of changing the wind speed ( $V_{\text{d}}$ ), coning angle ( β ) and the pitch angle ( θ ) on the flight performance ( T and Ω ) of the natural samara. From this, we hope to extract valuable insight and shed light on why samaras adjust their flight parameters within a particular range. The rest of the paper is as follows: the natural samara specimens, experimental rig set-up, and wind tunnel test procedures are described in §2. Section3 presents the results and insights obtained from the wind tunnel test. Lastly, concluding remarks from this study are presented in §4.

## 2. Description of the experimental rig and wind tunnel testing

Over recent years, several experimental rigs have been designed to study various features and forces produced by biological and artificial revolving wings [36–39]. All these rigs share similar mechanisms and principles in that they implement a motor to provide the rotary motion and use water or oil to match the operating Reynolds number (Re) as in nature, allowing for a larger wing size or slower flow speed. Contrary to these traditional rigs, the experimental rig presented in the current study offers a new novelty—it allows the testing of natural samaras in a similar airflow environment as in nature, while also facilitating testing outside their natural operating conditions. Furthermore, the rig allows the rotor to operate in a normal autorotation state (uncontrolled rotor speed) with near zero friction.

In light of this, the following section presents the novel experimental samara rig and details the wind tunnel test set-up. The section begins by introducing the three samara specimens and the novel *samara hub*, which is a specially designed rotor hub that can control the pitch and coning angle of the samara. Subsequently, the vertical wind tunnel and the *samara rig* are presented. The vertical wind tunnel provides an environment where autorotating seeds can be tested at various wind speeds and the samara rig enables the vertical thrust and rotational rate produced by the samara to be measured. Features of the samara rig, including the strain load cell and the frictionless rotation system are also presented. The section concludes with details of the experimental testing procedures and the post-processing of the obtained wind tunnel test signals.

### 2.1. Samara seeds

**Figure 3.**
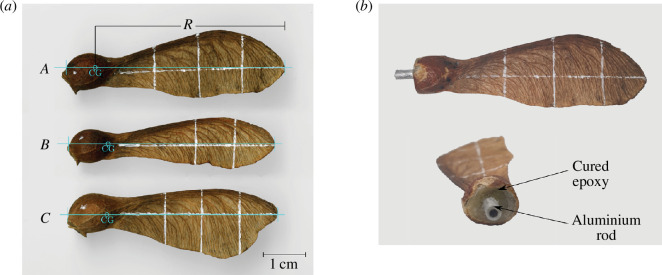
Sycamore seeds selected for the wind tunnel test. (*a*) Sycamores A, B and C in their natural form. CG denotes the determined location of the CG of each samara seed and *R* is the wing radius, i.e. the distance from the centre of rotation to the wing tip. (*b*) Modified sycamore A in preparation for the wind tunnel test.

The samara species chosen for the wind tunnel test was *Acer pseudoplatanus*, also known to us as sycamore seed. The seeds were collected during the peak of the dispersal season (autumn) in Brandon Hill Park, Bristol, UK. Sycamores that were seemingly immature, underdeveloped or had broken wings were excluded, and the seeds collected were stored in an air-tight plastic bag at room temperature prior to the testing. Three prime samaras, labelled herein as sycamore A, B and C, were selected for the wind tunnel testing. These seeds were selected from a group of sycamores (a total of nine) that were previously droptested in a different study, which brought forth the white reference lines that are visible on the samara wing [20]. The three seeds were specifically chosen to have appreciable differences in planform and wing area, to explore the effects of coning and pitch for three different blades. The wing planform and the CG location of each seed, estimated using the Plumb line technique [12], are shown in figure 3*a*. The flight parameters of the sycamore seeds recorded in the droptest, along with their geometrical properties in their natural form, are shown in table 1. Sycamore A was the individual with the lowest descent rate ( $V_{\text{d}}=0.97$ m s^−1^), followed by sycamores B and C ( $V_{\text{d}}=0.98$ and 1.08 m s^−1^, respectively). Vertical thrust produced by the autorotating samara in the droptest was assumed as T=mg , where m is the mass of the seed and g is the gravitational acceleration.

**Table 1.** Geometrical properties of Sycamore A, B and C in their natural form and the flight parameters of the autorotating samaras recorded in the droptest [20].

properties/parameters	sycamore A	sycamore B	sycamore C
mass, *m* (g × 10^−3^)	232	185	260
wing radius, *R* (cm)	4.47	3.82	3.97
wing area, *S*_w_ (cm^2^)	4.99	3.43	4.63
aspect ratio, AR	3.88	4.37	3.35
descent speed, *V*_d_ (m s^−1^)	0.97	0.98	1.08
vertical thrust, *T* (mN)	2.28	1.81	2.55
rotational rate *Ω* (rev min^−1^)	857	1304	957
coning angle, $\beta \;{(}^{\circ })$	19.4	15.1	8.6

To control the coning and the pitch angles, the seeds had to be modified and mounted on a specially designed rotor hub referred to as the *samara hub*. The nut end was first sanded down to a flat surface, opening up a chamber where the seed was located. The seed was partially removed, and any hollow space remaining was filled with epoxy resin. Once the epoxy had set, a small metal link rod was attached to the end of the sanded surface. This rod acted as a link to the samara hub. The final configuration of the modified sycamore A can be seen in figure 3*b*.

### 2.2. Samara hub

**Figure 4.**
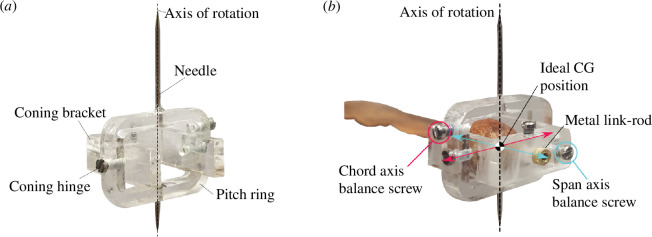
The *samara hub* (*a*) elements and (*b*) mass balancing system.

The *samara hub* was designed to allow the same samara seed to be tested at different flight settings. The miniature hub was built out of acrylic and consisted of two parts, the U-shaped *coning bracket* and the rectangular *pitch ring*, as shown in figure 4*a*. The main frames were produced via laser cutting and joined together by a set of micro-screws. The modified samara was installed by inserting the metal link rod within the coning bracket and locked in place via a dedicated pitch lock screw. The coning bracket controlled the β angle of the samara wing via the coning hinge. The coning hinge was designed to be stiff so that the prescribed β can be maintained throughout the experiment. By contrast, the rectangular pitch ring was held stationary, providing a reference baseline for changes in β and θ angles. The pitch ring also had two needles embedded within to effectively create a vertical axis. This axis acted as the axis of rotation, whereby the samara and the hub revolved around it as a single body. The samara was carefully positioned such that the CG of the natural seed was as close to the axis of rotation as possible. This ensured that the geometrical properties (wing radius, aspect ratio) that are influential to the aerodynamic performance were kept comparable to the droptest conditions.

The total mass of the samara hub was 0.962 g; around four times heavier than the natural samara. Thus, the samara was considered as having a heavy mass added to the nut end. The extra mass did not present complications as the objective of the experiment was to compute the vertical thrust produced by the samara wing. However, the heavy mass meant that the rotating system was no longer in static balance. During preliminary tests, it was found that the static balance was critical in achieving optimal autorotation; with poorly mass-balanced samaras showing interrupted and reduced rotational rate and lower thrust [20]. To address this issue, the samara hub was designed to have its own mass balancing system, whereby two balance screws could be screwed in and out, such that the CG position could be adjusted in the spanwise and chordwise direction, as seen in figure 4*b*. Prior to the experiment, the combined CG of the samara and the hub system was adjusted such that it lay approximately at the axis of rotation. The preciseness of the mass balance was checked by spinning the samara. If the samara and the hub were properly mass-balanced to a precise level, the samara would be able to spin for a prolonged time and could settle to a stop at any position within its disk of rotation. Furthermore, the samara hub was designed to have a small area to minimize the influence of drag.

### 2.3. Setting the coning and the pitch angle

**Figure 5.**
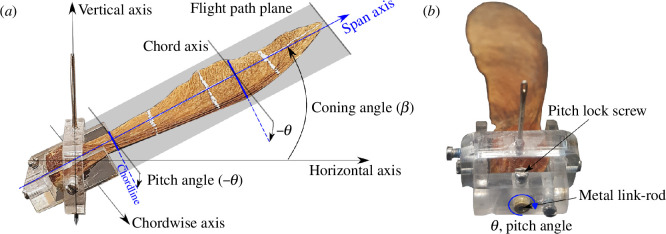
The experimental samara hub. (*a*) Graphical illustrations of the coning, β , and pitch, θ , angles of the samara, (*b*) set-up of the pitch angle of the samara.

It is important to state how the coning and the pitch angles were defined in the wind tunnel test. Graphical illustrations of β and θ of the samara wing can be viewed in figure 5*a*. β is the angle between the horizontal axis in the plane of rotation^1^ and the span axis. Positive β was defined as being the wing flapped up. θ is the angle between the chordline of the samara cross-section and the chord axis within the flight path plane. Positive θ was defined as being leading edge pitched up. With its paper-thin wing, the samara wing also exhibits an undulating twist along its span, which is drastically different for each seed. Hence, to acquire a more consistent measurement and controlled setting of the blade pitch, the datum for θ was set at the wing root of the samara ( ≈10%R ), where the level of twist is at its minimum. However, it should be noted that, in general, the twist may have significant implications on the spanwise thrust variations, as the blade twist will directly influence the local angle of attack along the wingspan. However, since our study focuses on investigating the effect of blade coning and pitch for the same samara wing, the impact of twist can be assumed to be non-dominant, at least for most of the testing conditions. It was anticipated, however, that testing at higher thrust conditions and relatively high wind speeds might induce larger elastic deformations of the wings, in which case the blade twist might have a more pronounced effect.

To adjust θ , the metal link-rod fixed to the samara was rotated manually and a dedicated pitch lock screw was used to lock the system at the desired θ , as seen in figure 5. In the experiment, we aimed to adjust θ in steps of −1.5° , ideally covering a range from 0 ° to −6.0 ° . To adjust β , the wing was simply flapped up manually to the desired coning angle. β was set in steps of 5 ° covering the range from 0 ° to 20 ° . It was estimated that the error in θ and β was <± 0.5 ° . Full details on the set-up of the pitch and coning angles of the samara are shown in appendix A.

### 2.4. Wind tunnel

**Figure 6.**
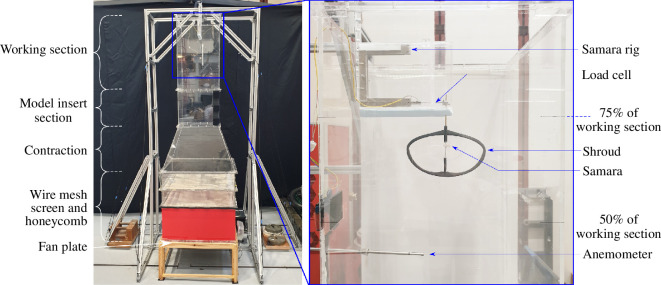
Overview of the samara wind tunnel test set-up.

The University of Bristol low-speed vertical wind tunnel is an open circuit wind tunnel with a contraction ratio of 3.2, capable of generating vertical upwards flow speeds ranging from 0.7 to 2.1 m s^−1^ at the working section. The schematics of the wind tunnel can be seen in figure 6. The square working section has dimensions of 34 cm × 34 cm × 60 cm, providing plenty of clearance space and minimal wall interaction to test autorotating seeds. A large part of the wind tunnel was built out of transparent Plexiglas, allowing clear observation of the samara in autorotation. The airflow within the wind tunnel was generated by nine computer specialized fans [40], all of which could be modulated concurrently under a single power source. Flow straightening and uniformity were achieved by installing a 20 cm thick honeycomb mesh and series of wire mesh separations positioned at the inlet of the wind tunnel. Real-time wind tunnel velocity was obtained using the TSI8455 air velocity transducer [41] mounted 15 cm downstream from the working section entrance. The velocity transducer offered an accuracy of ± 2 % of the measured reading and a resolution of 0.07 % of the full-scaled selected range (0−5 m s^−1^).

To characterize the flow quality, detailed wind tunnel characterization was conducted, as shown in appendix B. It was found that the flow at the working section was of exceptional quality; recording local mean velocities and turbulence intensities that varied by <2% and <1% , respectively, within a circular region of 13 cm in radius measured at the centre of the working section.

### 2.5. Samara rig and wind tunnel test set-up

The wind tunnel test set-up can be seen in figure 6. The idea was to constrain the autorotating seed to a fixed location within the working section and subject it to an upward flow of varying velocity in the range of 0.7 ∼ 2.1 m s^−1^. To hold the seed, a C-shaped platform, known as the *samara rig*, was developed. Other than housing the samara, the rig was designed to measure the vertical aerodynamic thrust (* T *) produced by the autorotating seed and its rotational rate ( Ω ) via the implementation of a strain load cell. The samara rig was positioned such that the autorotating samara lay approximately halfway between 50 and 75 % of the height of the working section, where the flow was at its most uniform, and the wind anemometer was placed at 25 % height of the working section to measure the wind speed upstream of the samara.

**Figure 7.**
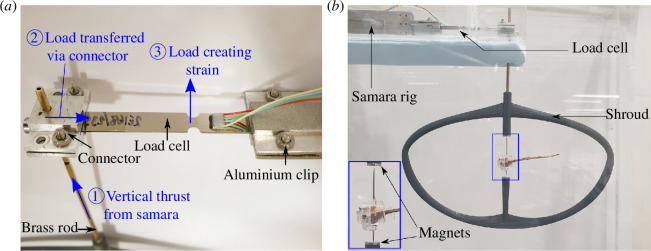
Elements of the samara rig. (*a*) Close-up view of the load cell and the connector intersection. The load path is highlighted in blue. (*b*) Shroud and minimal friction rotation system.

Figure 7*a* shows the elements of the samara rig in greater detail. The samara rig possessed a clip mount that could clamp one end of the load cell as a cantilever beam. At the other end of the load cell, precisely at the loading point, a brass rod was secured in place via a connector. The opposite end of the brass rod was interlinked to an oval-shaped shroud that accommodated the rotating seed. The load path from the seed to the load cell is as follows: the thrust produced by the seed was channelled into the oval shroud, transferred up the brass rod, and transmitted into the load cell as a vertical force, resulting in a strain.

The 16 cm × 9.5 cm × 0.3 cm sized shroud, shown in figure 7*b*, was designed primarily to house the samara. The shroud was required to be lightweight, rigid, and aerodynamic to minimize any interference with the flow. Furthermore, the closed-loop design of the shroud helped in damping out vibrations induced by the incoming flow and from the aerodynamic imbalance generated by the samara. The leading and trailing edges of the shroud were built with NACA0016 aerofoil cross-sections to minimize the generation of turbulent wakes and to provide enough structural rigidity. At the centre of the shroud, two Φ 6 mm magnets were set 3 cm apart, effectively creating a vertical-axis magnetic field. This set-up allowed the samara rotor hub to stand upright within the magnetic field and rotate about the vertical axis of rotation. It is important to note that only the rotor hub’s sharp needle end made contact with the bottom magnet throughout the experiment, accomplishing near-frictionless rotation. Sycamore seeds produce force in the micro-Newton scale while operating at high rotational rates; hence, minimizing the influence of friction was the critical factor in obtaining accurate measurements.

### 2.6. Strain load cell and characterization

Typically, sycamore seeds weigh around 200 mg and therefore produce approximately $2\times 10^{-3}\;N$ of vertical thrust in still air descent conditions ( $V_{\text{d}}\approx 1$ m s^−1^). To measure such a minuscule force, the load cell had to be of low capacity, highly sensitive, display a high level of resolution, and guarantee unerring accuracy and precision. Given these prerequisites, the *S100 thin film load cell* [42] with a maximum load capacity of 0.2 N was selected. The load cell is a miniature strain gauge that electronically displays changes in the strain due to force via a Wheatstone bridge configuration. The load cell signal was acquired using the *NI9237 strain and bridge input module* [43], which reconstructed signal with 24-bit resolution, a sampling rate of 25.6 kHz, and with a high bandwidth with zero inter-channel phase delay. As a result, the analogue load cell signal was instantaneously reconstructed with a resolution of 1.19 $\times 10^{-8}\;N$ .

To establish the relationship between the strain of the load cell and the force applied, detailed load cell characterization was conducted, as shown in appendix C. The characterization confirmed that the strain load cell produced an almost perfectly linear strain–load relationship with minimal hysteresis. Furthermore, it was estimated that the maximum combined error (drift, non-repeatability, nonlinearity) could be as great as $3\times 10^{-5}\;N$ . This error magnitude could be considered minor, amounting to around 1.5% of the thrust produced by the sycamore in natural descent $\left(2\times 10^{-3}\;N\right)$ .

### 2.7. Wind tunnel test procedure and signal processing

**Figure 8.**
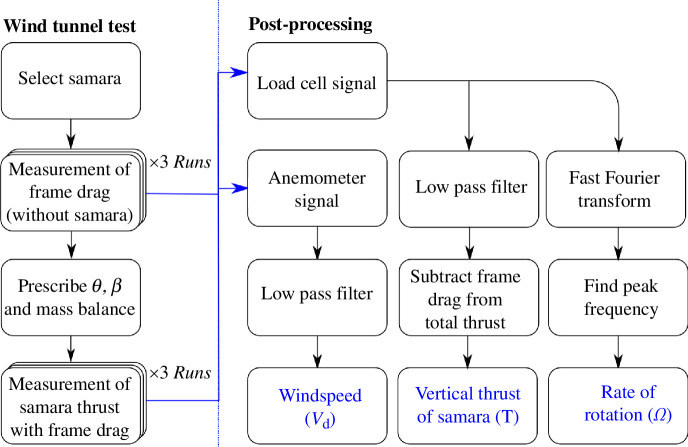
Flow chart illustrating the wind tunnel test procedures and the post-processing of the measurements.

The flow chart in figure 8 illustrates the procedures of the wind tunnel tests and the post-processing technique used to obtain the experimental data. Having selected the samara specimen, the first procedure was to conduct three wind speed *run*s of the wind tunnel test without the samara and the hub put in place. This was to isolate and measure the influence of the drag of the shroud at various wind speeds. Here, a *run* refers to the systematic increase in the wind speed from 0.73 to 2.10 m s^−1^ in a total of 15 steps, where each step represents an ≈ 0.09 m s^−1^ increment in the wind speed. At each specific wind speed, the load cell and the wind anemometer signals were recorded for a period of 30 s. Testing wind speeds of below 0.73 m s^−1^ or above 2.10 m s^−1^ was not conducted owing to the relatively higher wind tunnel flow turbulence, as mentioned in appendix B. Secondly, the selected specimen was installed within the samara hub at the prescribed θ and β angles. The samara hub system was also mass balanced both inside and outside the wind tunnel to ensure static mass balance around its axis of rotation. Subsequently, the samara hub was positioned within the testing rig, and three *run*s of wind tunnel tests were conducted with the autorotating samara in operation. A waiting time of 40 s was observed prior to recording the signal to ensure that the samara settled into steady autorotation before taking measurements.

The obtained load cell and anemometer signals were then post-processed. The static component of the load cell signal comprised the average strain created by the vertical thrust of the autorotating samara, combined with the drag of the shroud. Therefore, a low-pass filter with a cut-off frequency of 2 Hz was applied to the raw signal to obtain the average strain and to suppress any high-frequency noise. Subsequently, the influence of frame drag measured at the preliminary stage was subtracted from the average strain calculated. Finally, using the strain–load relationship established previously, the vertical thrust produced by the autorotating samara was deduced. Similarly, a low-pass filter with a cut-off frequency of 2 Hz was applied on the raw anemometer signal to obtain the average windspeed within the working section of the wind tunnel.

The dynamic component of the load cell signal comprised the cyclic change in the samara’s vertical thrust owing to its rotation and additionally, the load fluctuations owing to flow turbulence and vibrations emanating from rig imbalance. Hence, Fast Fourier Transform (FFT) was applied to the raw load cell signal. By identifying the peak frequency, the rotational speed of the samara was estimated with a resolution of 2 rpm. Full details on the signal processing can be viewed in appendix D.

### 2.8. Test matrix

**Figure 9.**
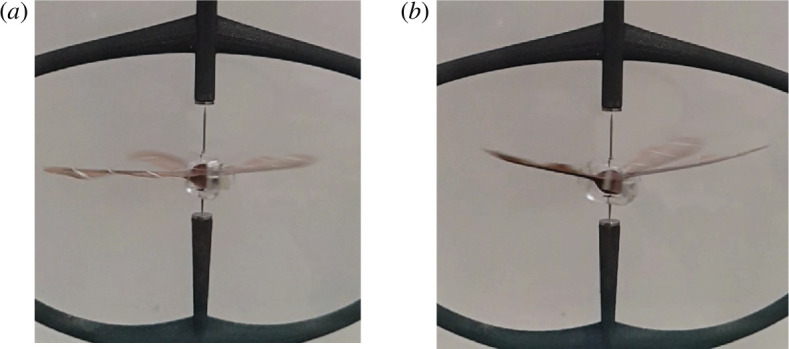
Sequential overlaid images of sycamore A spinning with (*a*) β=10° and (*b*) β=20° .

The following section describes the test matrix for sycamore A, which was similarly adopted for sycamores B and C. To start with, sycamore A was fixed at the pitch angle of θ=−2.5° and at β=0° . At these settings, the full testing procedure described in §2.7 was carried out. After three wind speed runs, the samara and the samara hub system were taken out and β was changed to 5° . Subsequently, the samara was restored onto the rig and tested systematically, covering the full coning angle range of β=0°,5°,10°,15° and 20° , while maintaining the same pitch angle. This β range allowed testing the seeds from zero degree coning case to the maximum achievable coning angle. Testing at β above 20° was not explored since the autorotating samara showed high levels of aerodynamic imbalance. Figure 9 shows the sequential images captured by a camera for sycamore A operating with β=10° and 20° . Once the data for the full coning angle range were obtained, the pitch angle of sycamore A was then changed to θ=−3.4° , and the above process was repeated. The authors aimed to adjust θ in steps of −1.5° , covering a range from 0 ° to −6° . However, since setting the θ was conducted manually, adjusting the pitch angle precisely proved to be difficult. Furthermore, the minimum pitch angle for each seed was limited by the minimum angle at which autorotation was possible. As for sycamores B and C, the experimental testing procedures were simplified considerably in that the effect of varying β was explored at a single θ and the effect of varying θ was studied at a fixed β of 10° . The full test matrix for sycamores A, B and C can be seen in table 2.

**Table 2.** Test matrix for sycamores A, B and C.

sycamore	velocity, *V*_d_	pitch angle, *θ*	coning angle, *β*
A	0.7 ∼2.1 m s^−1^	−2.5^◦^, −3.4^◦^, −6.0◦, −7.7^◦^	0^◦^, 5^◦^, 10^◦^, 15^◦^, 20^◦^
B	0.7 ∼2.1 m s^−1^	−0.7^◦^, −1.0^◦^, −1.7◦, −4.5^◦^ for β = 10^◦^	0^◦^,5^◦^,10^◦^,15^◦^,20^◦^ for θ =−1.0^◦^
C	0.7 ∼2.1 m s^−1^	−1.1^◦^, −1.5^◦^, −2.3^◦^, −4.0^◦^, −6.2^◦^ for β = 10^◦^	0^◦^, 5^◦^, 10◦, 15^◦^, 20^◦^ for θ =−1.5^◦^

## 3. Results

The following section displays the results and post-analysis of wind tunnel tests. The section begins by presenting the flight performance of sycamore A with changing wind speed ( $V_{\text{d}}$ ) and flight conditions ( β and θ ). The experimental data are then post-analysed to evaluate the effects of coning and pitch angles and from this, new insights into samara flight behaviours are deduced. Finally, to explore whether analogous results are reproduced for other samaras, wind tunnel test results for sycamores B and C are presented and discussed.

### 3.1. Flight performance of sycamore A

**Figure 10.**
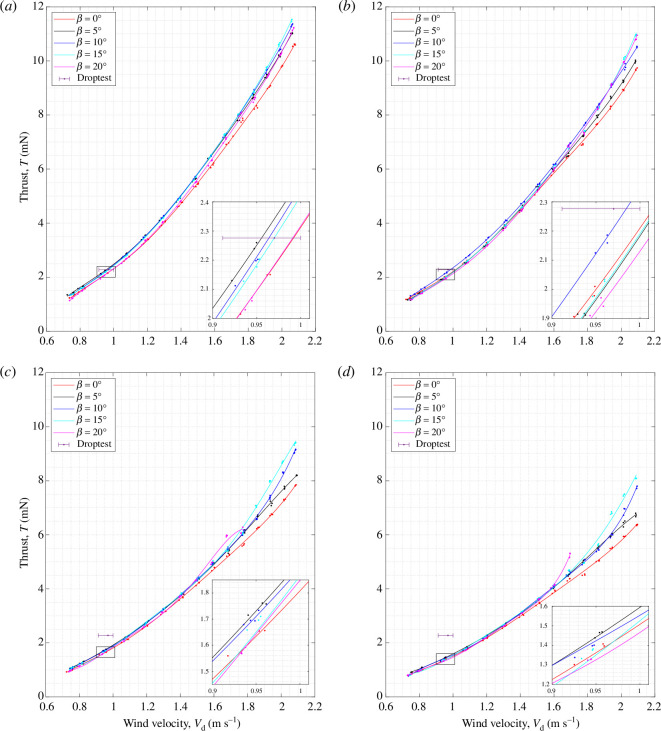
Variations in the vertical thrust, T , with the wind speed, $V_{\text{d}}$ , for sycamore A with varying coning angles, β and pitch angles of θ = (*a*) −2.6° , (*b*) −3.4° , (*c*) −6.0° and (*d*) −7.7° .

**Figure 11.**
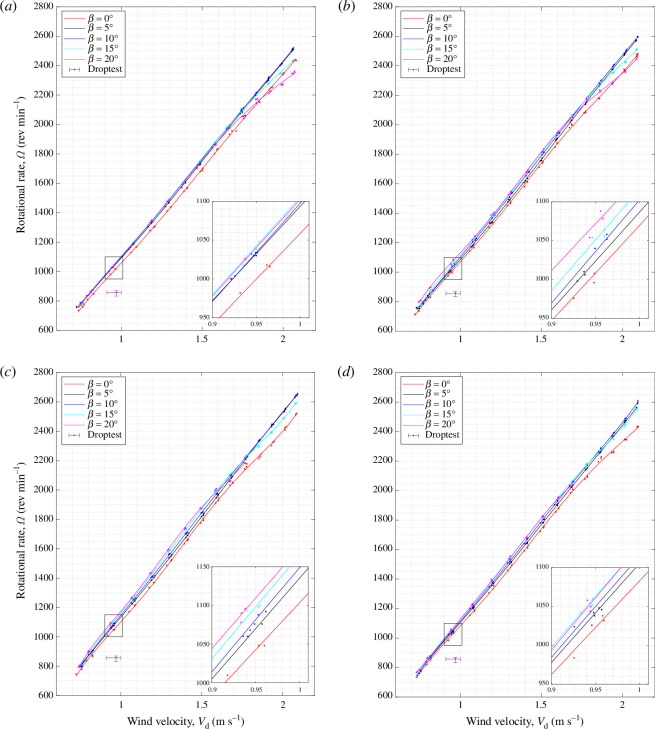
Variation in the rotational rate, Ω , with the wind speed, $V_{\text{d}}$ , for sycamore A with varying coning angles, β and pitch angle of θ = (*a*) −2.6° , (*b*) −3.4° , (*c*) −6.0° and (*d*) −7.7° .

Figures 10 and 11 show the vertical thrust, T , and rotational rate, Ω , produced by sycamore A at a range of wind speeds from $V_{\text{d}}=0.7∼2.1$ m s^−1^, and with varying coning angles from β=0° to 20° . Each figure contains four subplots, with each subplot corresponding to sycamore A operating at different pitch angles of θ=−2.6°,−3.4°,−6.0° and −7.7° . Pitch setting of θ=−2.6° was the lowest pitch angle achievable with the samara wing struggling to sustain steady autorotation for θ>−2.6° . For cases of high pitch angles ( θ=−6.0° and −7.7° ) accompanied with relatively high wind speed conditions ( $V_{\text{d}}>1.8$ m s^−1^), sycamore A exhibited high levels of lateral vibrations owing to aerodynamic imbalance. This created unwanted additional strains on the load cell, resulting in increased levels of T as observed in figure 10. To better showcase the data trends in figures 10 and 11 lines of best fit of fifth-degree polynomials were plotted for the T and Ω experimental data points. The best-fit lines were based on the least squares fit method employed in the MATLAB function ‘polyfit’.

Using the comprehensive set of experimental data generated, one can extract valuable insights into the flight behaviour of samaras outside their natural descent conditions. For instance, it can be revealed from figures 10 and 11 that $T\propto V_{\text{d}}^{2}$ and $\Omega \propto V_{d}$ . These trends are not explicitly shown here but are illustrated in appendix E with corresponding fitting relationships. Despite the fact that the bulk of the results follow a second- and first- order polynomials for T and Ω , respectively, the authors decided to keep the fifth-order polynomial fitting to capture any higher-order trends owing to nonlinearity in the aerodynamics and the effects of twist or bending deformation that can occur at relatively high loading conditions.

Furthermore, before carrying out further analysis, it is important to validate whether the experimental data results are reflective of the natural samara flight. Therefore, droptest results of sycamore A, as referred in table 1, were overlaid in figures 10 and 11 for comparison. One can see that the best match in the flight performance manifests at θ=−2.6° , where the discrepancies in T recorded for wind tunnel tests and droptests are less than 0.2 mN at $V_{\text{d}}=0.97$ m s^−1^. As for Ω , the discrepancies can be considered relatively large at the same $V_{\text{d}}$ , with the droptest recording an Ω=847 rpm in comparison to Ω=1000∼1020 rpm recorded for the wind tunnel test.

The authors believe that these differences in the flight performance originate from the dissimilarities in the test conditions. The operating pitch angle of sycamore A in the droptest was estimated to be close to 0° , as referred to in a previous study [12]. Hence, pitch setting of θ=−2.6° adopted in the wind tunnel test is similar but different, leading to a close but unequal flight performance. Nonetheless, the closeness of the results proves that the wind tunnel tests give a true representation of the autorotating samara and can measure the T and Ω of the natural samara to a high degree of accuracy.

### 3.2. Effect of coning angle

At first glance of figure 10, the change in coning angle appears to have little effect on the vertical thrust of sycamore A. All curves appear to be on top of each other, showing very little discrepancies in T . This outcome suggests that the role of β could be trivial, merely being a byproduct of establishing equilibrium in the moment about the flapping axis; a balance between the aerodynamic force, which pushes the wing tip upwards, and the centrifugal force combined with the wing weight, which pushes the wing down. However, if one observes the zoomed plot of figure 10, adjusted around the natural descent speed of the sycamore ( $V_{\text{d}}=0.9∼1.0$ m s^−1^), it is noticeable that having a moderate coning angle of β=5∼15° can increase the vertical thrust produced, and this phenomenon is observed for all the explored pitch angles.

The above outcome is surprising since it initially contradicts our understanding [44]. If one assumes that the samara wing is producing steady thrust during autorotation, increasing β should decrease the vertical thrust produced since it would effectively lead to a smaller disk area. Furthermore, introducing higher β will tilt the generated aerodynamic lifting force (assumed normal to the wing surface), which would further reduce the vertical thrust component. This statement was confirmed by the current authors using a numerical model based on the Blade Element Momentum theory, where an autorotating samara with constant thrust-generating capability (i.e. constant lift curve slope) generated lower T and higher Ω with increasing β [12]. However, the findings in figure 10 seem to present a different story.

**Figure 12.**
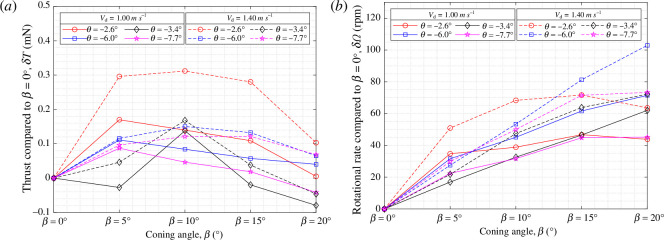
Variation in the (*a*) vertical thrust, δT , and (*b*) rotational rate, δΩ , relative to the values obtained for β=0° , presented for $V_{\text{d}}=$ 1.0 and 1.4 m s^−1^.

To further clarify the effects of β , the resulting data in figures 10 and 11 were re-represented in figure 12 as plots showing the changes in thrust ( δT ) and rotational speed ( δΩ ) relative to the values obtained at β=0° . δT and δΩ were evaluated at two specific wind speeds of $V_{\text{d}}=$ 1.00 and 1.40 m s^−1^, of which the former corresponds to the natural descent speed of the samara and the latter constitutes the samara wing operating at higher wind speeds. It is clear that moderate β is beneficial for sycamore A, with a majority of the peaks in δT occurring at β=5° and 10° for both wind speeds. The most pronounced δT manifests at θ=−2.6° , where δT reach values of 0.17 and 0.31 mN for $V_{\text{d}}=$ 1.00 and 1.40 m s^−1^, respectively. These values are equivalent to 7.3% and 6.5% increase in T compared with the values at β=0° . However, if too much of a coning is present, as in β=20° , the vertical thrust produced can become less than that of β=0° . It is also noticeable that sycamore A autorotates with an increased Ω with a higher β setting. For nearly all the cases, the maximum Ω manifests at β=20° . Whether this trend will continue at higher coning angles requires further investigation.

**Figure 13.**
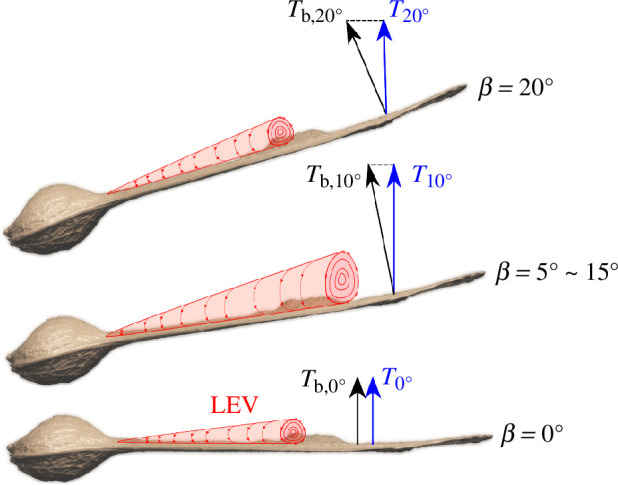
Illustration of how LEV changes with the coning angle and the implications on the net thrust produced by the samara. $T_{\text{b}}$ is the thrust perpendicular to the flight path plane, and T is the vertical thrust perpendicular to the horizontal axis. Note that the figure depicts a hypothesis, and future experiments are needed for validation.

The above results revealed that the vertical thrust and rotational rate increase with the coning angle up to a certain value. To explain this observation, the authors hypothesize that the size and strength of the LEV could be becoming greater with β up to a certain value. Previous studies on revolving and flapping wings have established that the LEV is generated and maintained by the balance between the spanwise pressure gradient, the centrifugal force and the Coriolis force. The interplay of these forces generates a spanwise axial flow within the LEV’s core [45–47], which keeps the LEV from growing too large. Correspondingly, substantial spanwise flow has been widely observed for revolving wings operating at Re in the $O(10^{3}∼10^{4})$ [48], including autorotating seeds [21–23], and this spanwise flow is said to play a major role in stabilizing the LEV for wings operating at such Re.^2^ Similarly, the coning angle, via its tilting of the wing relative to the horizontal axis, could be redirecting a portion of the incoming flow (from below) as a spanwise flow across the wing and thereby encouraging the formation of a stronger LEV. Thus, with a higher β setting, an enhanced LEV could be manifesting, as illustrated in figure 13. However, if the β angle becomes too high ( β>20° ), the effect of smaller disk area and tilt of the thrust vector becomes too dominant, leading to a reduction in the vertical thrust component. In addition, the strength of the LEV might also get weaker beyond β>20° , but it is not possible to confirm this statement since the experiment was not conducted at higher angles. Altogether, this may be the reason why sycamores and single-winged autorotating seeds tend to operate with a coning angle between 10° and 20°.

If higher β leads to a stronger LEV, it could also explain the higher Ω observed. For autorotating samaras, the rate of rotation is predominantly regulated by the balance between the ‘driving’ and ‘driven’ autorotative torques. The driving torque is produced at the inboard region of the samara wing, where the resultant force vector at each spanwise blade section is tilted forwards with respect to the vertical axis, owing to high operating inflow angles, ϕ=60°∼80° [12,16]. Conversely, the outboard region (area near the wingtip) with much lower operating ϕ=5°∼15° , produces a trailing resultant force and hence generates a resistive torque that needs to be ‘driven’ [20]. This ultimately means that the LEV, with its absence of stall mechanism, will influence the in-board region at a greater degree compared with the outboard region (i.e. the wingtip). Therefore, a stronger LEV will predominantly intensify the inboard thrust and the driving torque, meaning that samara must spin faster (i.e., operate at a higher Ω ) to achieve a new balance in the torque. Although not shown here, previous work by the authors using a numerical model for autorotating samaras based on the Blade Element Momentum theory showed that higher lift slope values (describing a stronger LEV) lead to higher vertical thrust and rotational rates [20], which matches the hypothesis.

The LEV hypothesis may not be the only explanation for the increase in T and Ω with β . During the wind tunnel flight experiment, it was observed that introducing moderate β (i.e., β=5° , 10° , or 15° ) greatly enhanced the dynamic stability in comparison to the case with β=0° and 20° . The sycamore rotated much more stably and smoothly with minimal flutter and lateral vibrations across all wind velocities defined. This may indicate that there could be a range of β angles that helps the sycamore to operate closer to its optimal autorotation and hence explain the reason why sycamore exhibits an increase in T and Ω with β up to a certain value.

### 3.3. Effect of pitch angle

**Figure 14.**
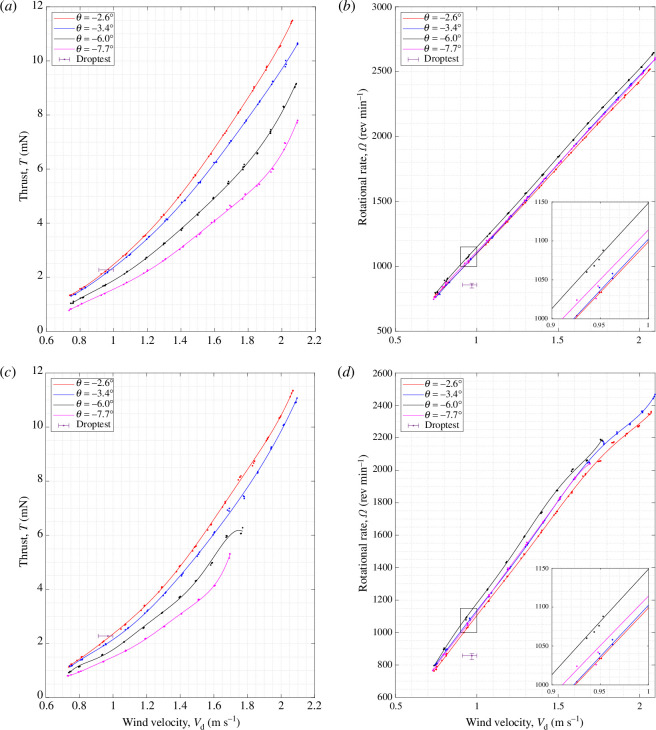
Effect of varying the pitch angle, θ , on the vertical thrust, T and rotational rate, Ω , for sycamore A operating at a fixed coning angle of (*a*,*b*) β=10° and (*c*,*d*) β=20° .

Looking at the results in figures 10 and 11, it can be seen that increasing θ to a more negative angle results in a reduced vertical thrust. To better visualize this effect, the wind tunnel test data were re-plotted as illustrated in figure 14, which shows the variations of T and Ω with varying θ for a range of wind speeds. The plots were generated for β=10° and 20° . It is clear that the vertical thrust reduces significantly with higher θ . Furthermore, the sensitivity of T is much more pronounced for changes in θ compared with β . For example, increasing the β from 0° to 10° leads to +0.31 mN in δT at $V_{\text{d}}=1.40$ m s^−1^ ( +6.5% ), whereas decreasing θ from −2.6° to −7.7° , results in a −2.0 mN in δT at the same $V_{\text{d}}$ ( −39.3% ). Hence, for autorotating samaras, θ is one of the most influential parameters that dictates the thrust-generating capabilities. In comparison, Ω seems very much insensitive to changes in θ .

Moreover, by comparing the wind tunnel results with the droptest flight data, one can deduce that sycamore A naturally operates with a pitch close to θ=−2.6° during descent in still air conditions. This finding is especially insightful since knowledge of the operational θ angle of natural samaras has been limited owing to the intricacies of measuring small angles. In fact, to the best of the authors’ knowledge, there is only a single study that has presented θ angles of samaras and it dates back to 1989, where Azuma & Yasuda documented that many samaras operate with θ that varies from approximately −1°∼−2° [8].

It is also worth noting that θ=−2.6° was the lowest achievable θ angle (manually set) within the wind tunnel tests of sycamore A. When the pitch angle was set to a value lower than −2.6° (i.e., closer to 0° ), the sycamore failed to autorotate. This suggests that a natural samara in descent will most likely operate precariously close to the autorotational boundary in θ to maximize the vertical thrust, resulting in a lower descent rate.

### 3.4. Flight performance of sycamores B and C

**Figure 15.**
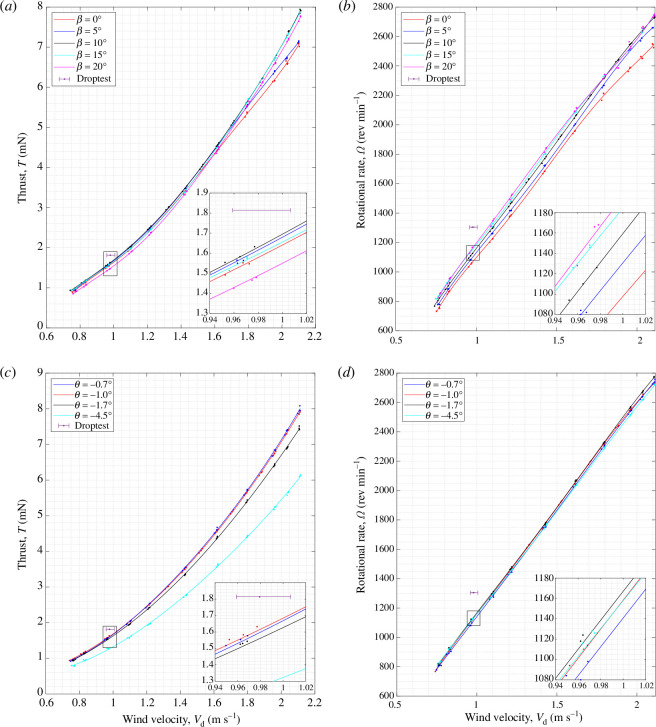
Flight performance of sycamore B, showing the variations in the vertical thrust, T and rotational rate, Ω , with wind speed, $V_{\text{d}}$ . The sycamore was operated with (*a*,*b*) varying coning angle and at a constant θ=−1.0° and (*c*,*d*) with varying pitch angle and at a constant β=10° .

**Figure 16.**
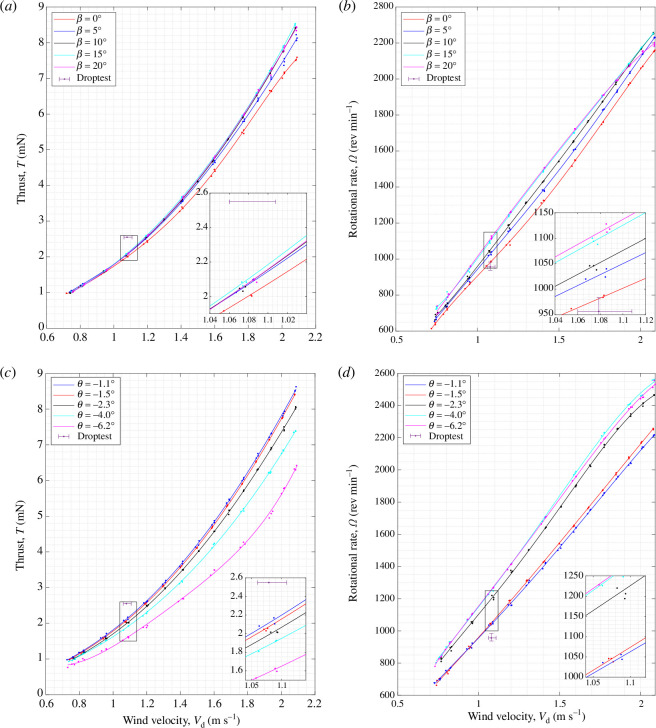
Flight performance of sycamore C, showing the variations in the vertical thrust, T and rotational rate, Ω , with wind speed, $V_{\text{d}}$ . The sycamore was operated with (*a*,*b*) varying coning angle and at a constant θ=−1.5° and (*c*, *d*) with varying pitch angle and at a constant β=10° .

To investigate whether the above outcomes can also be obtained for other samaras, wind tunnel tests were carried out for sycamores B and C, and the results are shown in figures 15 and 16, respectively. Looking first at the general trends, there is a clear relationship that $T\propto V_{\text{d}}^{2}$ and $\Omega \propto V_{\text{d}}$ for both sycamores B and C, analogous to the results of sycamore A.

As for the coning angle, figures 15*a* and 16*a* show that increasing β has a positive effect on the vertical thrust, with the peak *T* manifesting at β=10° and 15° , respectively, for sycamores B and C at $V_{\text{d}}=1.00$ m s^−1^. figures 15*b* and 16*b* also show that higher β gives an increased Ω for both sycamores, and the maximum Ω rates are recorded at β=20° . As for the pitch, the lowest θ attainable was θ=−0.7° and −1.1° , respectively for sycamores B and C. These θ angles were also the pitch settings that produced the highest vertical thrust, as illustrated in figures 15*c* and 16*c*. Increasing θ negatively also resulted in a high reduction in T for both sycamores. On the other hand, very little discrepancies in Ω were observed for the changes in θ for sycamore B, as seen in figure 15*d*. However, as for sycamore C, increasing θ negatively resulted in a higher rotational rate. The reason behind such discrepancies in the results remains unclear, but the current authors believe that this could be either owing to the effects of blade flapping or owing to small errors introduced while manually setting the blade pitch of the samara wing.

When comparing against the droptest results, the closest match occurred at θ=−1.0° for sycamore B, with the wind tunnel test producing approximately 0.3 mN and 270 rpm less T and Ω at $V_{\text{d}}=0.98$ m s^−1^. As for sycamore C, the closest match was obtained θ=−1.1° , where approximately 0.5 mN lower T and 100 rpm higher Ω were measured for the wind tunnel testing at the natural descent speed of sycamore C ( $V_{\text{d}}=1.08$ m s^−1^).

**Figure 17.**
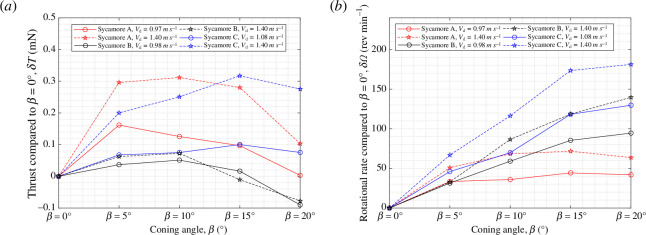
Variation in the (*a*) vertical thrust, δT , and (*b*) rotational rate, δΩ , relative to the values obtained for β=0° , presented for $V_{\text{d}}=$ natural descent speed and $V_{\text{d}}=1.4$ m s^−1^ for sycamores A, B and C. Pitch angles were set at θ=−2.6° , −1.0° and −1.5° , respectively, for sycamores A, B and C.

Additionally, a plot that summarizes the changes in the thrust ( δT ) and rotational rate ( δΩ ) relative to the values obtained for β=0° was generated for all three sycamores, as displayed in figure 17. The effect of β was evaluated at $V_{\text{d}}=$ natural descent speed and at $V_{\text{d}}$ = 1.40 m s^−1^. Figure 17*a* shows that a moderate coning angle is advantageous for the samara, with all the thrust peaks manifesting between β = 5 °∼15° . The greatest δT=0.32 mN is produced by sycamore C operating at $V_{\text{d}}=$ 1.40 m s^−1^. This equates to a 9.6% increase in T in comparison to β=0° . Adopting β = 20 ° seems unfavourable for the samara, with many showing a high reduction in δT . It is also noticeable that the coning angle at which the peak thrust occurs is different for each sycamore and changes with the wind speed.

As for figure 17*b* , all three sycamores showed an increase in δΩ with β . However, for many, increasing β from 15 ° to 20 ° gave a minimal change in δΩ and in some instances, outputted a negative δΩ . These results, as previously mentioned, could be owing to the samara LEV diminishing or the samara moving away from its optimal state of autorotation with high β angles.

**Figure 18.**
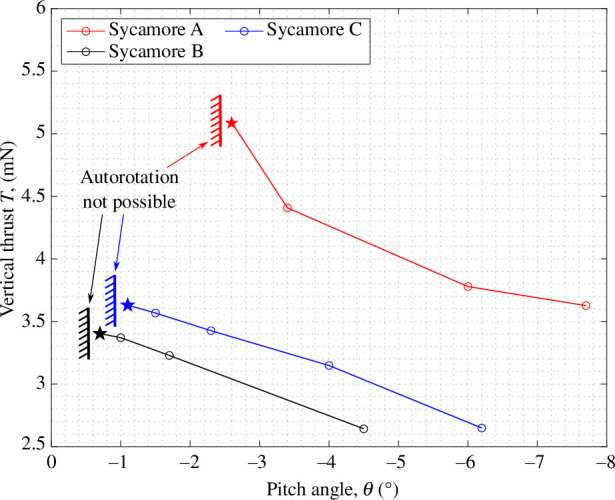
Variations in the vertical thrust, T , with pitch angle, θ , at $V_{\text{d}}=1.40$ m s^−1^. The stars denote the measured maximum thrust points, occurring at the lowest pitch angle to achieve autorotation.

Finally, figure 18 shows the variations in the T with θ for all three sycamores at $V_{\text{d}}=1.40$ m s^−1^. Stars indicate the lowest possible pitch angle setting attainable for each sycamore during the wind tunnel tests. The lowest pitch setting, defined by the autorotational boundary, was obtained by attempting to spin the rotor at progressively lower pitch angles in flow-on conditions. When the samara wing was unable to initialize autorotation or sustain it after an initial transient, it was deemed that autorotation was not possible. From the plot, it is clear that points of lowest pitch angle produces the maximum thrust. These results again support the hypothesis that the samara keeps the pitch as low as possible to maximize the thrust generating capacity.

As a final note, the discussions in this paper are presented in terms of vertical thrust maximization at a given wind speed. For a maple seed, however, the more natural target would be to minimize the descent speed for a given seed weight. Nonetheless, this analogy holds true, since in most of the flight envelope (as illustrated in figures 10 and 14), there is a monotonous (almost quadratic) trend between vertical thrust and descent rate. This confirms that there is an equivalence (though nonlinear) between vertical thrust maximization and descent speed minimization.

## 4. Conclusions

We conducted a series of wind tunnel tests to investigate the influence of flight parameters and wind speed on the autorotational performance of single-winged samaras. The species of interest were *Acer psuedoplatanus*, also known to us as sycamore seeds. In total, three individual seeds were tested. To adjust the flight parameters, each samara seed was installed within a bespoke rotor hub which enabled the control of the samara wing’s coning and pitch angle. The seed and the rotor hub were installed within a novel experimental rig that effectively allowed the samara wing to autorotate with minimal friction. A miniature strain load cell was integrated within the rig to record the vertical thrust and rotational rate produced by the samara wing. The rig was placed within the vertical wind tunnel that was specially designed for testing samara seeds. The wind tunnel test subjected samara wings with pre-configured pitch and coning, to a range of wind speeds from 0.7 to 2.1 m s^−1^, and measurements of the vertical thrust and rotational rate of the autorotating seed were collected.

This study successfully generated a novel and comprehensive set of experimental data of autorotating samaras with measurements of vertical thrust and rotational rates of the samara with changes in the wind speed and flight conditions. The originality of this study also consisted of using a special artificial hub, as it has allowed us to vary parametrically the coning and pitch angles of the samara wing, thereby generating complimentary information to the droptest data available in the literature. The analysis of the experimental data revealed new insights into samara aerodynamics. Firstly, it was observed that the thrust showed quadratic growth and the rotational rate displayed linear growth with increased wind speed for the autorotating samara. Secondly, it was discovered that introducing moderate coning angles between 5° and 15°, generated a higher vertical thrust that could be up to 9.6% greater compared to a samara without a coning angle. An increase in the rotational rate was also observed with a moderate coning angle. Thus, the coning angle was revealed to play a role in slowing down the descent of the samara. The authors hypothesized that this could be owing to the LEV getting stronger with a moderate coning angle. Additionally, it was found that samaras operate with negative pitch angles close to zero degrees from a range of approximately −0.7°∼−2.6° . These pitch angles are precariously close to the autorotational boundary and it was indicated that samaras adopt these low pitch angles to maximize the production of vertical thrust. The findings of this investigation would help to pave the way for the design of samara-inspired Unmanned Air Vehicles.

Future work to further consolidate the results obtained in this study would be to conduct PIV tests to visualize the changes in the strength and size of the LEV of samaras with varying coning and pitch angles. This would allow us to understand how single-winged samaras use the LEV to maximize their performance. Furthermore, obtaining wind tunnel test data for a larger collection and greater diversity of samaras would be valuable in revealing a more complete understanding of samara aerodynamic characteristics.

## Figures and Tables

**Figure 19.**
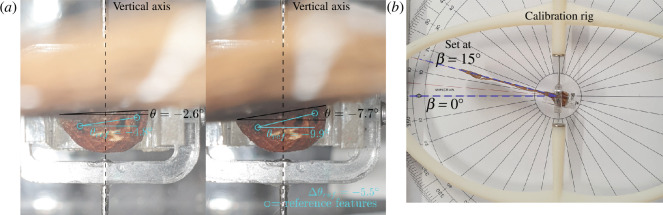
Set-up of the pitch and coning angles of the samara. (*a*) Photogrammetry technique used to establish the pitch angle setting at the root of the samara wing: left, sycamore A at the lowest pitch, θ=−2.6° ; right, at the highest pitch, θ=−7.7° . (*b*) Coning calibration rig.

**Figure 20.**
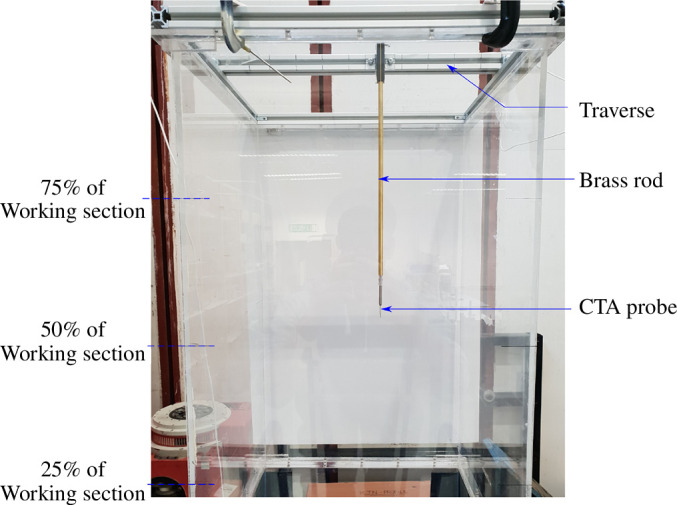
Photograph of the wind tunnel characterization process. The hot wire probe is currently measuring at 50% height of the working section. Longer and shorter lengths of brass rods were used to measure at 75 and 25% height of the working section.

**Figure 21.**
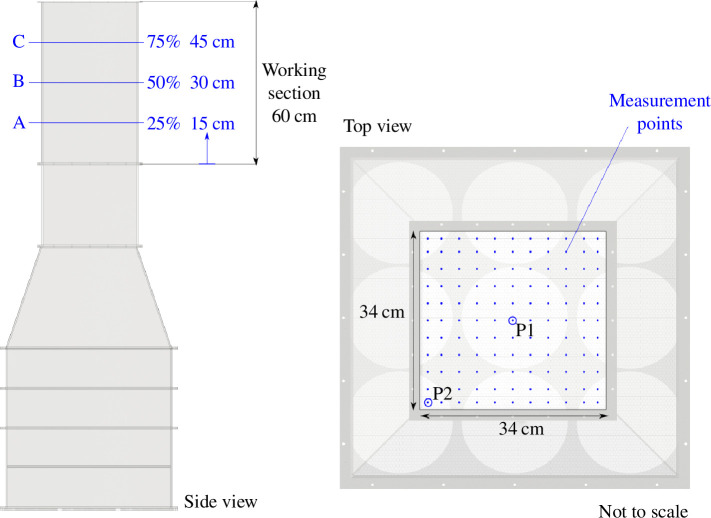
Illustration of the side view and top view of the wind tunnel. Left diagram shows the three height planes that the characterization took place on the working section and the right diagram shows the measurement points across the working section, conducted for each plane. P1 and P2 are defined as the centre and corner points, respectively.

**Figure 22.**
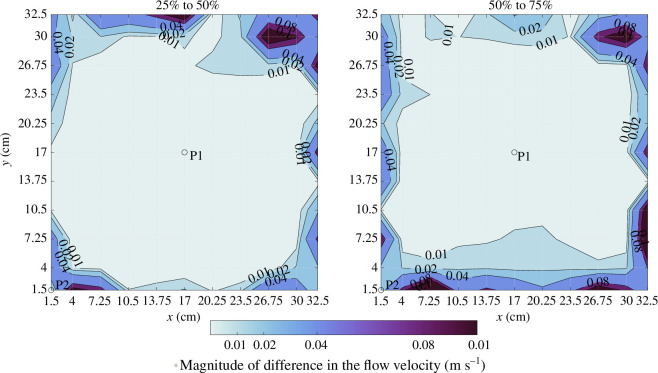
Difference in the mean flow velocities between 25– 50% and 50– 75% heights of the working section.

**Figure 23.**
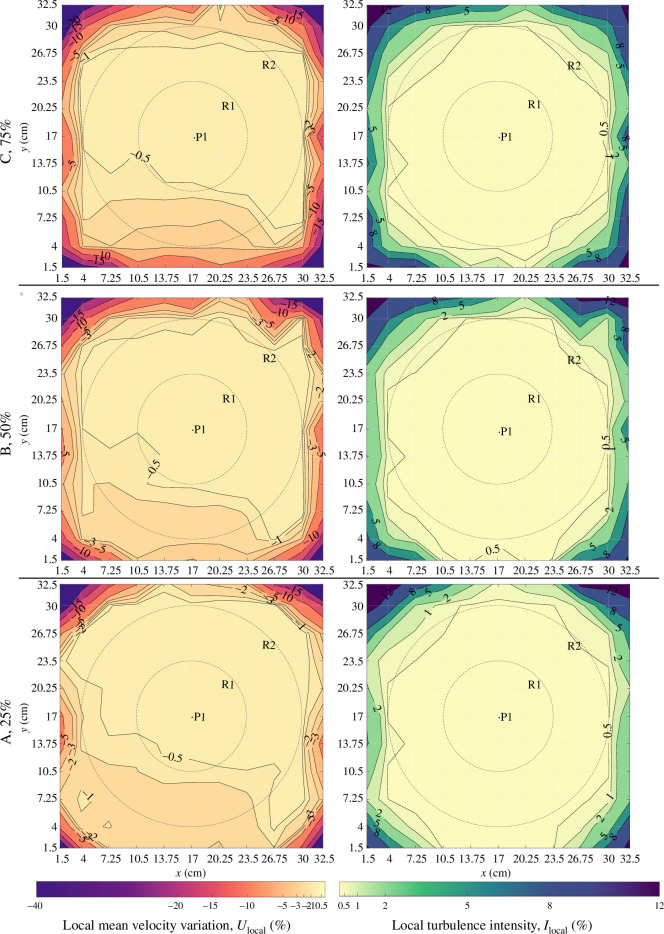
Local mean velocity variation ${\bar{U}}_{local}$ and local turbulence intensities $I_{local}$ across the working section at the heights of 25, 50 and 75% . The flow was fixed at around 1.57 m s^−1^ at the centre point, *P*1. *R*1 is the experimental region for natural samara testing and *R*2 is the larger experimental region.

**Figure 24.**
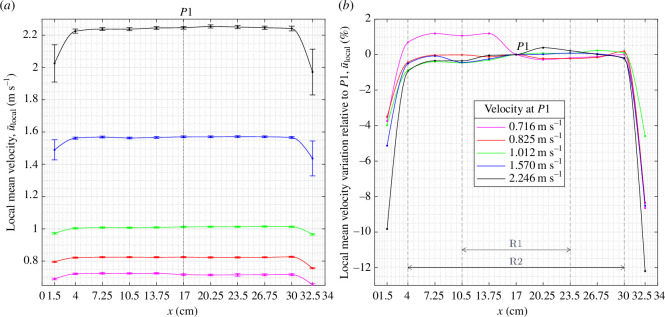
Graphs showing the local mean velocity ${\bar{u}}_{local}$ (*a*), and the local mean variation relative to centre point P1 ${\bar{U}}_{local}$ (*b*), for five different velocity settings. The local mean velocities at the centre point, P1 , range from 0.716 to 2.246 m s^−1^. For the local mean velocity profile graph, the root mean square of the flow, $u_{rms,\;local}$ , are plotted on top as error bars.

**Figure 25.**
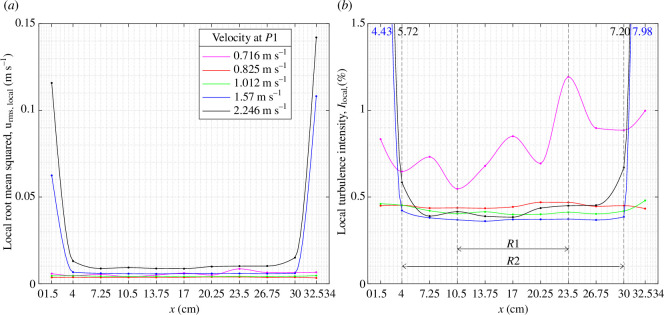
Local root mean squared, $u_{rms,local}$ (left) and turbulence intensity, $I_{local}$ , (right) for five different velocity settings.

**Figure 26.**
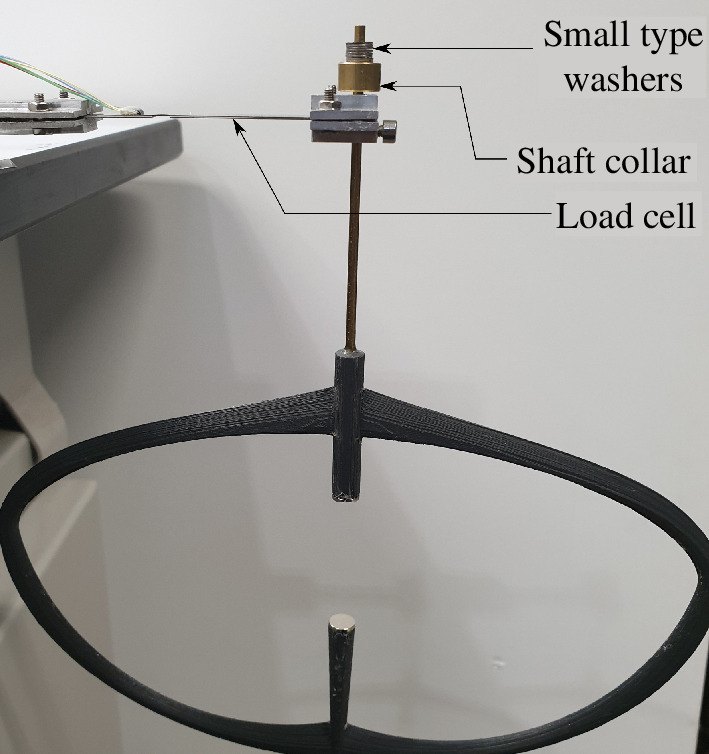
Load cell characterization set-up. Either a set of small or large washers were loaded onto the load cell.

**Figure 27.**
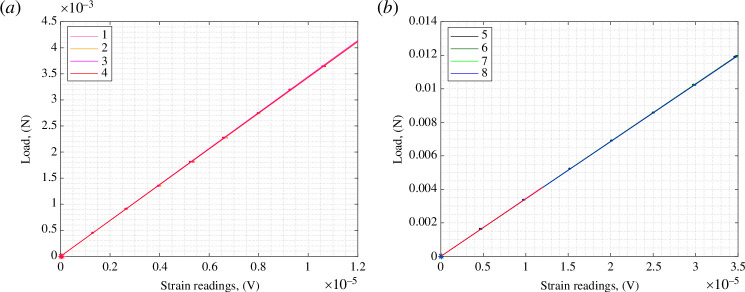
Load cell characterization graph with (*a*) small and (*b*) large washers. First-order, best-fit line that intercepts the origin was drawn for each of the four repeats.

**Figure 28.**
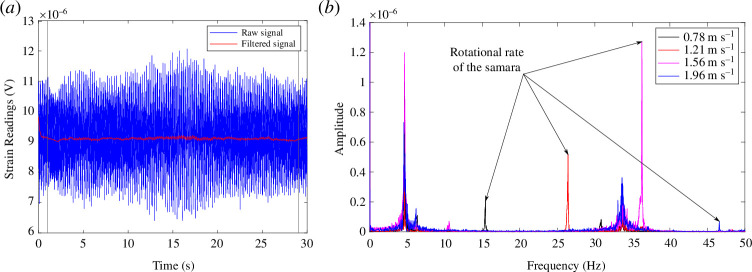
Post-processing of the strain load cell signal. (*a*) Strain reading ( V ) with time ( t ) of the load cell signal at constant wind speed. The first and the last second of the signals were omitted from the static mean strain readings. (*b*) Overlaid FFT of load cell signals at four different flow velocities.

**Figure 29.**
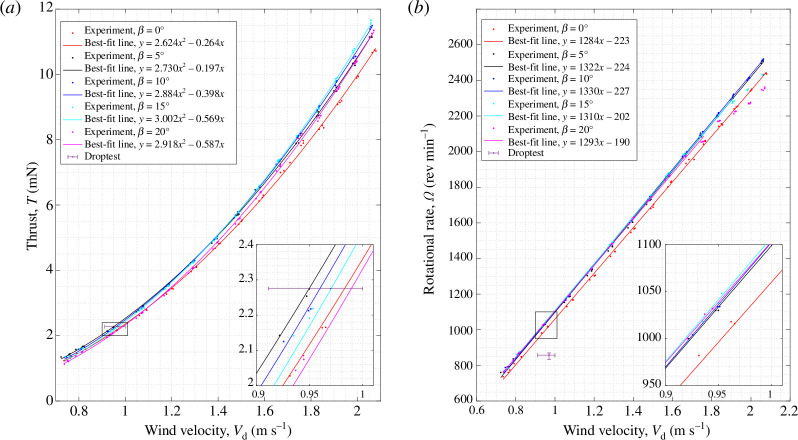
Variations in (*a*) the vertical thrust, T , and (*b*) rotational rate, Ω , with the wind speed, $V_{\text{d}}$ , for sycamore A with varying coning angles, β and constant pitch angle of θ=−2.6° .

**Figure 30.**
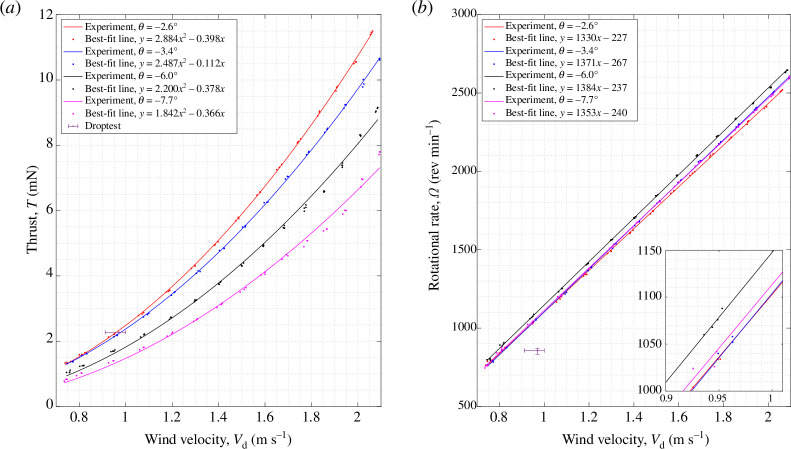
Variations in (*a*) the vertical thrust, T , and (*b*) rotational rate, Ω , with the wind speed, $V_{\text{d}}$ , for sycamore A with varying pitch angles, θ and constant coning angle of β=10° .

## Data Availability

All data is available online via Dryad [49].

## Footnotes

^1^
The plane of rotation is defined as the plane normal to the vertical axis of rotation.

^2^
It should be noted that the spanwise flow is not always the source of LEV stability. While strong spanwise axial flow has been clearly evident for flapping wings operating at Re of $O(10^{3}∼10^{4})$ , little spanwise flow has been observed at fruit fly scale Re of $O\left(⩽10^{2}\right)$ [48].

## Appendix A. Pitch and coning angle set-up

This section of the appendix details the set-up of the pitch and coning angles of the samara. To set the pitch angle ( θ ), the metal link rod fixed to the samara was rotated manually and a dedicated pitch lock screw was used to lock the system at the desired θ angle. In the experiment, we aimed to adjust θ in steps of −1.5° , ideally covering a range from 0° to −6.0° . Since θ adjustment was conducted manually, setting the pitch angle accurately proved to be difficult. Therefore, it was decided to use photogrammetry to establish the exact changes in the θ manifested after each modulation. Figure 19*a* shows the photograph of the underside of sycamore A at the lowest and highest θ settings. One can see that the pitch at the near root of the wing could be extracted. However, since the chordline of the wing is not entirely clear, it was deemed that extracting the pitch directly via inspection could introduce significant errors. Therefore, using two reference features on the samara nut, which are indicated by cyan circles in figure 19*a*, the changes in the relative pitch angle, $\Delta \theta_{ref}$ , were calculated, and added to determine the absolute pitch angle. The total error estimated in measuring θ was estimated to be <±0.5° .

To set the coning angle, β , the samara hub and the samara were placed within a coning calibration rig, as seen in figure 19*b*, and the samara wing or the coning bracket was simply flapped up manually to the desired coning angle. β was set in steps of 5° covering the range from 0° to 20° ° Despite the procedure being manual, the calibration rig significantly reduced the error in the β set-up and the authors estimate the errors to be <± 0.5°.

## Appendix B. Wind tunnel characterization

This section of the appendix details the procedures and the results of wind tunnel characterization. To characterize the flow quality, a StreamLine Pro constant temperature anemometer (CTA) system [50] equipped with a 55P16 wire probe [51] was used to measure the cross-sectional velocity profile at three different heights (25, 50 and 75 % ) of the working section. Prior to testing, the hot-wire probe was calibrated using a Dantec 54H10 calibrator, and data were captured at a sampling frequency of 86kHz using the NI9215 module. Together, the CTA system was able to accurately measure both laminar and turbulent flows with less than 0.4% relative error and 0.002 m s^−1^ absolute error in flow velocity.

Figure 20 shows the hot wire placed within the working section of the wind tunnel. The probe was set up to be precisely in line with the flow for accurate measurement, and longer or shorter brass rods were used to measure at 25, 50 and 75% height of the working section. A manually driven traverse controlled the longitudinal and the lateral position of the probe. At each height, the cross-sectional velocity profile was obtained by recording the flow velocity at 121 measurement points (11 × 11) orderly spread across the 34 cm × 34 cm working section, as seen in figure 21. For each measurement point, 10s of flow was measured, accounting for around 855,000 samples of flow velocity, and via equation (B 1), the local mean velocity was calculated.

(B 1)
$${\bar{u}}_{local}=\frac{1}{N}\sum_{i=1}^{N}u_{i},$$

where ${\bar{u}}_{local}$ is the local mean velocity at each specific measurement point, *i* and *u_i_* are the sample number and the sample velocity and N is the total number of samples.

The strength of the turbulence at each measurement point was indicated by the root mean square of the flow. The root mean squared of the flow can be calculated by the equations (B 2), (B 3). $u_{rms}$ or the root mean squared of a flow represents the standard deviation of the ‘random’ velocity fluctuations; the higher the $u_{rms}$ , the higher the level of turbulence in the flow:

(B 2)
$$u\prime_{i}=u_{i}-{\bar{u}}_{local},$$

(B 3)
$$u_{rms,\;local}=\sqrt{\frac{1}{N}\sum_{i=1}^{N}(u\prime_{i}{)}^{2})}.$$

**Table 3.** Flow velocity at the centre points (P1) and corner points (P2) at 25, 50 and 75% heights of the working section.

height	$\bar{u}$ _P1_, (m s^−1^)	$\bar{u}$ _P2_, (m s^−1^)
25%	1.5712	1.1441
50%	1.5699	1.165
75%	1.5687	1.1365

To look at all 121 measurement points across the cross-section and to compare values via sensitivity, the above two flow parameters were normalized. This introduced two new parameters. The first parameter is the local mean velocity variation relative to the centre point ( ${\bar{U}}_{local}$ ), which can be calculated using equation (B 4):

(B 4)
$${\bar{U}}_{local}=\frac{{\bar{u}}_{local}}{{\bar{u}}_{\text{P}1}}\times 100.$$

This normalizes the mean velocity relative to the centre point, P1. To measure the turbulence, a second parameter, the local turbulence intensity can be calculated using equation (B 5). This normalizes the mean turbulence or the $u_{rms,\;local}$ relative to the local mean velocity:

(B 5)
$$I_{local}=\frac{u_{rms,\;local}}{{\bar{u}}_{local}}\times 100.$$

For the characterization, the first investigation was to see whether the flow velocity was analogous at the centre (P1) and corner points (P2) at different heights of the working section. Table 3 shows the mean velocities at P1 and P2 at different heights of the working section with the wind tunnel operating at a fixed wind speed of approximately 1.57 m s^−1^. One can see that the flow is smooth at the centre, with almost identical flow velocities of around 1.57 m s^−1^ recorded at the centre points, P1. However, the flow velocity is reduced and inconsistent at the corner points, P2, indicating a less stable flow owing to wall interactions.

To get the complete picture of the cross-section of the working section, figure 22 was plotted, which is a contour map showing the magnitude of difference in the mean flow velocities between the 25– 50% heights and the 50– 75% heights of the working section. The contour maps shows that there are less than 0.01 m s^−1^ differences in flow velocity across the majority of the working section with a change in height, suggesting that the flow is steady within the working section. Only near the walls, can one observe greater change in the flow speed.

The second investigation aimed to assess the flow quality at each specific height of the working section. Similarly, the wind tunnel was operated at a fixed wind speed of 1.57 m s^−1^. Figure 23 shows the flow quality ( ${\bar{U}}_{local}$ and $I_{local}$ ) at 25, 50 and 75% heights of the working section. For comparative purposes, a reference circular region of 6.5 cm, covered by the autorotating natural samara, was outlined as R1. Additionally, a larger experimental region of 13cm radius was drawn and outlined as R2.

Looking first at the local mean velocity variations, it can be seen that ${\bar{U}}_{local}$ are within −2∼0.5% of the centre velocity for the majority of measurement points at all three heights. In fact, within the region of R1, the flow quality is exceptional with ${\bar{U}}_{local}<\pm 0.5\%$ , implying that the *R*1 region is a suitable area to test natural samaras. Even with a much bigger region of R2, the ${\bar{U}}_{local}$ are within −2∼0.5% of the centre velocity. However, one must be careful in experimenting too close to the walls of the wind tunnel; especially near the corners where mean velocities have reduced by more than −20% of the centre velocity.

As for local turbulence intensities, $I_{local}$ , all three heights show near identical contours. Generally within the regions of R1 and R2, the measured $I_{local}$ are less than 0.5% , 1% , respectively. Only near the corners, can one find turbulence intensities that develop over 12% . As a result, one can conclude that the flow at the working section is of exceptional quality at a wind speed of 1.57 m s^−1^; recording local mean velocities and turbulence intensities that vary by <2% and <1% , respectively within a circular region of 13 cm radius measured at the centre of the working section.

The last investigation explored the flow quality of the working section with a change in flow velocity. In total, five different wind speeds were explored, ranging from 0.716 to 2.246 m s^−1^ when referenced at the centre point, *P*1. Rather than examining the whole cross-section, the spanwise velocity profile at the midcross-section of the working section at 50% height was investigated, as seen in figure 24.

Figure 24 shows that all five velocity settings have similar spanwise local mean velocity profiles. It shows a flat plateau from the centre point to the sides of the working section, *x* = 4 cm and *x* = 30 cm, with very little change in the local mean velocity. However, near the walls, the local mean velocity decreases, and this reduction is greater for higher speeds. This finding, at first glance, suggests that between 4 and 30 cm of the working section, (equivalent to *R*2 region) good flow quality is established at all wind speeds defined.

For further inspection, the local mean velocities were normalized relative to the centre point velocity for all five wind speeds, as seen in figure 24. The graph shows that for all but one wind speed, ${\bar{U}}_{local}$ are within −1 to 0.5% within the *R*1 and *R*2 regions. However, for the lowest wind speeds of 0.716 m s^−1^, ${\bar{U}}_{local}$ measured is above 1% within the *R*2 region. This was owing to the fans working intermittently as there was insufficient electrical power for all the fans.

Figure 25 shows the local root mean squared of the flow, $u_{rms}$ (the level of turbulence), at five different wind speeds defined. One can see that the turbulence strength is low across the span of the working section for the lower three wind speed settings. For higher wind speeds, 1.570 and 2.246 m s^−1^, $u_{rms}$ does increase but it is still under 0.015 m s^−1^ within the *R*2 region. Near the walls, there is a dramatic increase in the $u_{rms}$ indicating the presence of turbulence and separation. Since turbulence scales with velocity, $u_{rms}$ was normalized with the local mean velocity to obtain $I_{local}$ . Within the region of *R*2, $I_{local}<0.6\%$ is maintained for the four wind speeds from 0.825 to 2.246 m s^−1^. However, for the lowest velocity setting (0.716 m s^−1^), $I_{local}$ is both fluctuating and higher than its counterparts across *R*2, suggesting that the flow is unstable. To conclude, the above findings suggest the flow has acceptable low turbulence and velocity variations within regions *R*1 and *R*2 for velocity settings of 0.825, 1.012, 1.570 and 2.246 m s^−1^.

## Appendix C. Load cell characterization

This section of the appendix details the procedures and results of S100 thin film strain load cell characterization. The objective behind the load cell characterization was to deduce the relationship between the strain of the load cell and the force applied—the strain load cell outputs the strain in Volts, hence the reading must be converted back into force in Newtons in order to evaluate the vertical thrust of the autorotating samara. Figure 26 shows the set-up of the load cell characterization, whereby metal washers of known mass were successively loaded and then unloaded, to produce a load–strain curve. The washers were loaded on top of a shaft collar such that their weight directly pushed the brass rod downwards, which consequently created a downward strain in the load cell. This set-up was analogous to the wind tunnel test—during the experiment, the load cell was always strained downwards owing to the weight of the shroud and the brass rod, even with the maximum vertical thrust produced by the autorotating samara.

Two types of metal washers were used for the load cell characterization: small and large. The small washers weighed approximately 0.045 g each, giving an increment of 5 × 10^–4^ N of force. A total of eight small washers were loaded to cover the experimental thrust range of the natural samara. The large washers weighed around 0.170 g, giving an increment of 1.8 × 10^–3^ N of force and up to seven large washers were loaded to reach a total load of up to 0.012 N. For both small and large washers, the characterization was repeated four times.

Figure 27 shows the load–strain curves produced by load cell characterization for small and large washers, respectively. The characterization confirmed that the strain load cell produced almost perfectly linear strain–load conversion relationship with minimal hysteresis. For each set of experimental data, a line of best fit of first order ( y=mx ) was drawn and the slope of the curves gave the conversion rate between strain and load. The values of the slope for all the load–strain curves are displayed in table 4.

**Table 4.** Slope and the drift for the load cell calibration with small and large sets of washers.

repeats	slope, ( $\frac{N}{V}$ , 10^2^)	drift in V , 10^−8^	drift in N , 10^−5^	% of drift relative to ^a^
1	3.426	8.87	3.05	1.52
2	3.434	5.48	1.88	0.94
3	3.451	4.31	1.48	0.74
4	3.434	1.52	0.52	0.26
5	3.432	5.29	1.81	0.91
6	3.433	8.06	2.77	1.38
7	3.423	8.19	2.81	1.41
8	3.422	2.95	1.01	0.50
avg	3.432	5.58	1.92	0.96

^a^ The amount of drift is compared with the benchmark thrust of which is the estimated thrust of the natural samara in vertical descent.

By taking an average of the eight repeats, it was deduced that:

(C 1)
$$\text{load}=3.4321\times 10^{2}\times \text{strain reading}.$$

Furthermore, it was estimated that the maximum combined error owing to drift, non-repeatability, and nonlinearity, could reach approximately 3 × 10^–5^ N, as seen in table 4. This magnitude in the error could be reviewed as being minor, amounting to around 1.5% of the thrust produced by the sycamore in natural descent (2 × 10^–3^ N).

## Appendix D. Signal processing

This section of the appendix details the post-processing of the strain load cell signal. Figure 28*a* shows the raw load cell signal produced by an autorotating samara operating at a constant wind speed, recorded for 30 s. The static component of the raw signal constitutes the average strain created by the vertical thrust of the autorotating samara, combined with the drag of the shroud. The dynamic component represents the cyclic change in the samara’s vertical thrust owing to its rotation, as well as the load fluctuations owing to flow turbulence and vibrations emanating from the rig imbalance. To extract the mean strain, a low-pass filter with a cut-off frequency of 2 Hz was applied to the raw signal to obtain the filtered signal, represented in a red colour line, and the average of the filtered signal within the time-frame of 28 s gave the mean strain; the first and last second were omitted owing to filter edge effect. Subsequently, the mean strain was converted into the vertical thrust via the conversion rate determined in load cell characteriation, and finally, the drag of the shroud was deducted.

To extract the samara rotational rate, FFT was applied to the raw load cell signal. Figure 28*b* shows the overlaid FFTs of the autorotating samara operating at four different specific wind speeds. One can clearly see the four distinct frequency peaks, each corresponding to the rotational rate of the samara at the given wind speed. The two coherent peaks at 5 Hz and 34 Hz, visible for all wind speeds, correspond to the frequencies originating from the cantilever boundary condition and the natural frequency of the load cell. All the peaks are singular and sharp, suggesting that the samara is autorotating stably, and the error in extracting the rotational rate was estimated to be equal to the FFT resolution of 0.033 Hz or 2 rev min^−1^, which accounted for less than 1 % of the lowest rotational rate measured within the wind tunnel test.

## Appendix E. Flight performance of sycamore A with revised lines of best fit

This section of the appendix presents the flight performance of sycamore A with revised lines of best fit. Figure 29 corresponds to revised plots of figures 10 and 11 within the main manuscript and (figure 30) corresponds to revised plots of figure 14*a,b*. Second-order polynomials were fitted for the vertical thrust and first-order polynomials were fitted for the rotational rate experimental points. Fitting relationships are also provided for all the polynomials drawn.
